# A Case of SAPHO Syndrome Complicated by Uveitis with Good Response to Both TNF Inhibitor and JAKinib

**DOI:** 10.1155/2023/6201887

**Published:** 2023-01-18

**Authors:** Ritasman Baisya, Meghna Gavali, Mudit Tyagi, Phani Kumar Devarasetti

**Affiliations:** ^1^Department of Clinical Immunology and Rheumatology, Nizam's Institute of Medical Sciences (NIMS), Hyderabad, India; ^2^Vitreoretinal Diseases, LV Prasad Eye Institute, MS Ophthalmology, Hyderabad, India

## Abstract

**Introduction:**

SAPHO (synovitis, acne, pustulosis, hyperostosis, and osteitis) syndrome is a rare autoinflammatory condition describing the constellation of inflammatory skin, bone, and joint manifestations which result in diagnostic difficulty and therapeutic challenge.

**Case:**

Here, we present a case of a young male diagnosed with SAPHO syndrome with osteoarticular and cutaneous involvement from an early age in his life. He suffered diagnostic challenges for a long time and was hence inadequately treated. He had minimal response to conventional DMARDs but showed excellent response to TNF inhibitor (adalimumab). Later, he defaulted treatment and presented with acute anterior uveitis which was also dramatically improved with adalimumab and tofacitinib although financial constraint was always an issue for the patient.

**Conclusion:**

The uniqueness of this case was that the patient had a multiorgan involvement including osteoarticular system, skin, and eye. Both TNFi (adalimumab) and JAKinib (tofacitinib) had a good response to all organs with a net improvement in the quality of life of this patient.

## 1. Introduction

SAPHO (synovitis, acne, pustulosis, hyperostosis, and osteitis) syndrome is a rare form of autoinflammatory condition involving cutaneous and musculoskeletal (bone and joint) systems. Owing to varied manifestations, it often poses diagnostic and therapeutic challenges [1]. First reported by Chamot et al. [2] in 1897, its osteoarticular symptoms include osteitis of the axial skeleton (spine, sacroiliac joints, and pelvic joints); inflammatory arthritis of knees, ankles, metacarpophalangeal (MCP), and metatarsophalangeal (MTP) joints; enthesitis. Dermatological features include acneiform eruption, palmoplantar pustulosis, hidradenitis suppurativa (HS), pyoderma gangrenosum (PG), Sweet's syndrome, and Sneddon–Wilkinson disease [3, 4]. Its prevalence is estimated at 0.11/100,000 in Caucasians and 0.00144/100,000 in Japanese with no preference for gender or age [5]. Skin involvement is reported in 85% of all patients and frequently manifests after osteoarticular symptoms [6]. Uveitis is a rare finding, but SAPHO syndrome can be considered a part of the spondyloarthropathy spectrum, and it seems logical to find the association of SAPHO with uveitis. Here, we have reported a case of SAPHO complicated with uveitis.

## 2. Case Presentation

**Figure 1:**
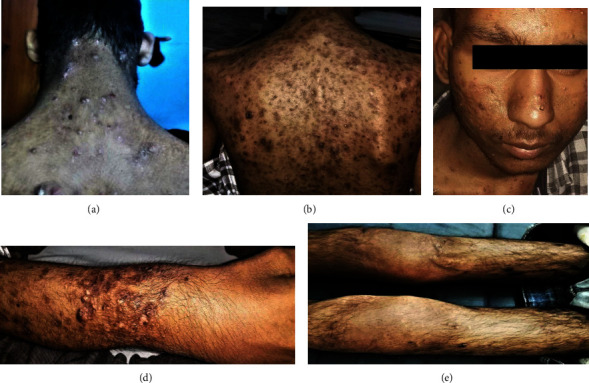
(a) Multiple pustular acne over back, (b) acnes healing with hyperpigmentation and scar, (c) few acnes with oily skin of face (d) pustular rashes over forearm with wrist synovitis and bony swelling, (e) active synovitis in knee with black pustules on leg.

**Figure 2:**
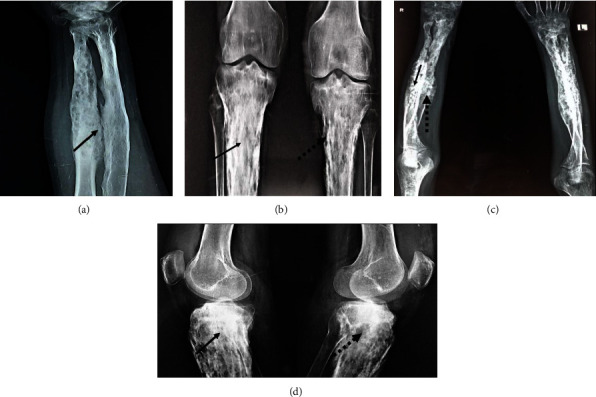
(a) X-ray right forearm (both radius and ulna) showing hyperostosis with few lytic area, (b) X-ray knee (AP) showed bilateral tibia periosteal reaction, hyperostosis, osteolysis with osteopenia, (c) Lytic lesions and hyperostosis increased after 1 year during replace, (d) increased sclerotic lesion, lytic lesion with osteopenia after 1 year during relapse. Plain arrow-sclerotic lesion, dotted arrow-lytic lesion.

An eighteen-year-old male, symptomatic for six years, had developed pain and swelling in bilateral forearms and had been diagnosed with chronic osteomyelitis following a bone biopsy. He subsequently noticed acneiform and pustular lesions over the face, which spread to the neck, trunk, and limbs that crusted and healed with residual hyperpigmentation. Initial skin biopsy showed inflammation with giant cells in the epidermis and lymphoplasmacytic infiltrate in the dermis with a probable diagnosis of tubercular verrucosa cutis. He was treated with antitubercular therapy (ATT) for six months without any improvement. Repeat skin biopsy showed nonspecific inflammation with dense neutrophilic infiltrate. Meanwhile, the patient developed inflammatory polyarthritis of both knees, ankles, and sternoclavicular joints when he visited our outpatient clinic. On examination, he had active synovitis in both knees, wrist, elbow and sternoclavicular joints, multiple acneiform and pustular lesions over the chest, back, face, and upper and lower limbs (Figure 1). Imaging of both knees and forearm showed evidence of subchondral hyperostosis with arthritis changes (Figure 2). Investigation showed that mild leukocytosis, raised ESR and CRP, antinuclear antibody by immunofluorescence, and HLA B27 by flow cytometry were negative. With clinical findings of acne vulgaris, synovitis, pustulosis, and radiologic findings of osteitis and osteomyelitis, he was diagnosed with a case of SAPHO syndrome. As no genetic association is reported in SAPHO, the diagnosis was made based on clinico-radiographic findings (as per Kahn and Benhamou criteria). He was started on nonsteroidal anti-inflammatory drugs (NSAID) and sulfasalazine (2 g/day) with minimal response even after 4 weeks. After a month, a TNF inhibitor (injection of adalimumab, 40 mg subcutaneously every 15 days) was started fortnightly. The patient had significant improvement in both osteoarticular and cutaneous symptoms by three months after six doses of adalimumab, but he defaulted to treatment.

**Figure 3:**
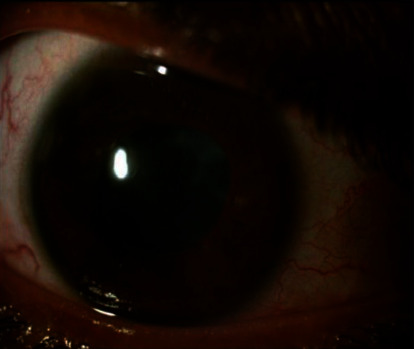
Showing uveitis in right eye complicated with posterior synechiae, poorly dilated pupil and complicated cataract.

Four months later, he developed acute onset right eye painful redness with the blurring of vision and was diagnosed as acute anterior granulomatous uveitis with posterior synechiae formation (Figure 3). He was treated with topical steroids and ophthalmic surgical correction, but low-grade inflammation was persisting in slit lamp examination. This was followed by osteoarticular and cutaneous flares. This time he had multiple crusted hemorrhagic plaque-like lesions over both legs along with severe acne and pustules. Biopsy of the plaque-like lesion revealed nonspecific inflammation with no evidence of vasculitis adalimumab was restarted resulting in a rapid improvement in articular, cutaneous, and ocular manifestations within two months. He was however noncompliant to treatment due to financial constraints which resulted in another cutaneous and articular flare three months later. Hence, he was shifted to jakinib (tofacitinib, 5 mg BD) and resolutions of symptoms achieved within short period. After use for three months, he defaulted again as he couldn't afford tofacitinib also. Hence, he has been shifted to adalimumab again with government health support, and he is doing well since the last visit.

## 3. Discussion

SAPHO syndrome should be suspected when a young patient presents with predominant large joint arthritis, axial arthritis, and osteomyelitis with certain cutaneous changes. Its diagnosis is based on clinic-radiological findings and after the exclusion of infective or malignant aetiology. There are a few criteria proposed for this entity, among them modified Kahn and Benhamou's criteria are commonly used. As per modified Kahn criteria [7], inclusion points include bone-joint involvement with PPP and psoriasis vulgaris or severe acne or chronic bowel disease, isolated sterile hyperostosis/osteitis in adults, chronic recurrent multifocal osteomyelitis in children after excluding infectious osteitis, tumoral conditions, and noninflammatory condensing lesions in the bone while Benhamou criteria [8] include (i) axial, sterile, recurrent multifocal chronic osteomyelitis with or without dermatosis; (ii) acute, subacute or chronic arthritis associated with palmoplantar pustulosis, pustular psoriasis or severe acne; and (iii) any sterile osteitis in association with palmoplantar pustulosis, pustular psoriasis, or severe acne. Making a diagnosis is challenging because not all symptoms are always apparent or present at the same time, or some may be subtle. Our patient had severe acne, pustulosis with sterile osteitis, and synovitis fulfilling the diagnostic criteria of SAPHO syndrome.

Other differential diagnoses should be ruled out before making a diagnosis of SAPHO (Table 1). The two most important differentials are infectious and neoplastic. Other autoinflammatory diseases also should be considered such as psoriatic arthritis, Paget disease, and Tietze syndrome. Regarding differential diagnosis in the paediatric age group, it includes Ewing's sarcoma, histiocytosis, Majeed syndrome (caused by recessive mutation in *LPIN2* gene), or deficiency of Il-1 receptor antagonist (DIRA) (caused by recessive inherited mutation in *IL1-RN* gene) [9].

Although the pathogenesis remains elusive, elevated levels of TNF-*α* in bone specimens of SAPHO patients have been described recently [10], and the use of the TNF-*α* blocking agents (infliximab and etanercept) has been reported with significant improvement.

Daoussise et al. [11] identified 36 articles of SAPHO syndrome treated with biologic and included in the analysis. A total of 64 cases were treated with biologics (44 with TNF blockers, 7 with IL-1 blockers, 12 with biologics targeting the IL-23/IL-17 axis, and 1 with tocilizumab). Data support a positive effect of anti-TNF treatment in SAPHO with a response rate in particular manifestations of 95.4%. Skin disease improved in 21/29 cases (response rate 72.4%). In SAPHO patients not responding to conventional treatment, TNF blockers should be the first choice. Most cases of severe/refractory SAPHO syndrome were treated with infliximab [12, 13] due to the long history of use with this agent. Adalimumab has been recently used in a few published case series with satisfactory reports. [14]. A review of adalimumab use in SAPHO mentioned that in the majority of cases (78%), there was a rapid response after 2–4 weeks of therapy and maintenance of clinical disease remission during the treatment period (ranging from 1 up to 42 months). [15] (Table 2)

Literature showed variable cutaneous response with TNF inhibitors mainly with infliximab. Several authors [16] reported a less predictable outcome of cutaneous disease manifestations, with worsening or reactivation of skin lesions as well as paradoxical psoriasiform eruptions during treatment of SAPHO syndrome with TNF inhibitors. In a case report by Arias-Santiago et al. [17], treatment with adalimumab showed excellent response to both articular and cutaneous manifestations while infliximab worsened the skin lesions in the same patient. In other case reports [18], adalimumab showed excellent cutaneous response along with osteoarticular improvement. In this case, the patient had significant improvement in cutaneous manifestation after using a TNF inhibitor.

Association of SAPHO syndrome and uveitis was first reported by Villaverde et al. [20] in a 40-year-old male patient with sacroiliitis, acne conglobata, and acute anterior uveitis. The author suggested that the SAPHO syndrome was part of the spondyloarthropathy spectrum, and uveitis might also be a common extra-articular manifestation of SAPHO syndrome. But very few cases of uveitis in SAPHO were reported subsequently. In a small case series by Takeda et al. [21], one male patient with SAPHO syndrome presented with acute anterior nongranulomatous uveitis. He was treated with topical steroids, methotrexate, and infliximab and showed significant improvement in his vision. In a single-centre study of 41 adult SAPHO patients by Aljuhani et al. [1], two patients had acute uveitis, and one with recurrent uveitis.

The use of JAK inhibitor in SAPHO syndrome is relatively new and limited to very few case reports. A 44-year-old woman with SAPHO was treated with tofacitinib 5 mg BD dose with marked improvement in arthralgia by 4 weeks [22]. Another case report of a 62-year-old female had significant improvement in cutaneous and articular manifestations after 4 weeks of tofacitinib [23]. Our patient had a good cutaneous and articular response after 1 month of tofacitinib use but could not afford the medicine due to financial constraints. The uniqueness of this case was that the patient had multiorgan involvement including eye, skin, and osteoarticular system. Both adalimumab and tofacitinib had a good response to all organs with a net improvement in the quality of life of this patient.

## 4. Conclusion

The combination of adalimumab with background conventional DMARD therapy proved to be safe and efficacious for the treatment of cutaneous, osteoarticular, and ocular involvement in SAPHO syndrome, determining the rapid onset and sustained clinical remission. Tofacitinib is another promising therapy although data are limited. Most of the published reports of SAPHO syndrome are individual case-based, so large, multicentre observational studies are needed in order to assess the long-term outcomes of patients affected by SAPHO syndrome.

## Figures and Tables

**Table 1:** Differential diagnoses of SAPHO syndrome in adults and children (infectious and malignancy excluded).

	Commonality	Difference
*In adults*		
Psoriatic arthritis	Axial, appendicular arthritis, and psoriasis	Dactylitis, nail changes, no acne, no osteomyelitis
Paget disease	Osteitis	Old age predominance, both lytic and sclerotic changes, no cutaneous involvement
Tietze disease	Clavicular involvement is seen	Lack of arthritis, cutaneous features
Sweet syndrome	Neutrophilic dermatosis and aching joint are presentations	Lack of axial and appendicular arthritis, no acne

*In children only*		
Majeed syndrome	Multifocal osteomyelitis	Congenital dyserythropoietic anaemia is typical of Majeed, transient skin changes, caused by recessively inherited mutations in *LPIN2*
DIRA(deficiency of Il-1 receptor antagonist)	Generalised pustulosis, osteitis, periostitis, and systemic inflammation	Recessively inherited mutation due to *IL1-RN*
Ewing sarcoma	Bony involvement	No skin changes
Histiocytosis	Bone and skin involvement	Clinical and pathological findings differ

**Table 2:** The comparison of different other studies with the present case report is depicted.

Study	Arias-Santiago et al. [17]	Vekic et al. [18]	Garcovich et al. [15]	Castellví et al. [19]	Present case
Patient	49 year female	26 year male	25 year male	28 year female	18 year male
Presentation	Axial involvement, PPP	Axial and peripheral arthritis, HS, PG	Severe acne, axial (SCJ, SI joint)	Axial with cutaneous (PPP)	Sterile osteitis, acne, PPP, uveitis
Biologic used	ADA	ADA	ADA	ADA	ADA
Response to ADA	Excellent response to cutaneous and osteoarticular	Improvement in both cutaneous and articular	Bony involve rapidly improved, acne improving with residual scarring, improved QOL	Both improved	Improvement in VAS, bony, cutaneous, and ocular symptoms
Other treatment	Infliximab—no response to cutaneous	Methotrexate	Methotrexate, isotretinoin	SAAZ,	SAAZ, methotrexate, later tofacitinib
Follow-up	10 month	9 months	24 months	18 months	Follow-up for 3 years—defaulted in between

PPP: palmoplantar pustular psoriasis, SAAZ: sulfasalazine, ADA: adalimumab, HS: hidradenitis suppurativa, PG: pyoderma gangrenosum, QOL: quality of life, SCJ: sternoclavicular joint, SI: sacroiliac joint, VAS: visual analogue scale.

## References

[1] Aljuhani F., Tournadre A., Tatar Z. (2015). The SAPHO syndrome: a single-center study of 41 adult patients. *Journal of Rheumatology*.

[2] Chamot A. M., Benhamou C. L., Kahn M. F., Beraneck L., Kaplan G., Prost A. (1987). Acne-pustulosishyperostosis-osteitis syndrome. Results of a national survey. 85 cases. *Rev Rhum Mal Osteoartic*.

[3] Marzano A. V., Borghi A., Meroni P. L., Cugno M. (2016). Pyoderma gangrenosum and its syndromic forms: evidence for a link with autoinflammation flammation. *British Journal of Dermatology*.

[4] Carneiro S., Sampaio-Barros P. D. (2013). SAPHO syndrome. *Rheumatic Disease Clinics of North America*.

[5] Beretta-Piccoli B. C., Sauvain M. J., Gal I. (2000). Synovitis, acne, pustulosis, hyperostosis, osteitis (SAPHO) syndrome in childhood: a report of ten cases and review of the literature. *European Journal of Pediatrics*.

[6] Levin J., Werth V. P. (2006). Skin disorders with arthritis. *Best Practice & Research Clinical Rheumatology*.

[7] Hayem G. Le syndrome SAPHO [SAPHO syndrome].

[8] Benhamou C. L., Chamot A. M., Kahn M. F. (1988). Synovitis-acne- pustulosis hyperostosis-osteomyelitis syndrome (SAPHO): a new syndrome among the spondyloarthropathies?. *Clinical & Experimental Rheumatology*.

[9] Rukavina I. (2015). SAPHO syndrome: a review. *Journal of Children’s Orthopaedics*.

[10] Przepiera-Będzak H., Brzosko M. (2021). SAPHO syndrome: pathogenesis, clinical presentation, imaging, comorbidities and treatment: a review. *Advances in Dermatology and Allergology*.

[11] Daoussis D., Konstantopoulou G., Kraniotis P., Sakkas L., Liossis S. N. (2019). Biologics in SAPHO syndrome: a systematic review. *Seminars in Arthritis and Rheumatism*.

[12] Olivieri I., Padula A., Ciancio G. (2002). Successful treatment of SAPHO syndrome with infliximab: report of two cases. *Annals of the Rheumatic Diseases*.

[13] Wagner A. D., Andresen J., Jendro M. C., Hulsemann J. L., Zeidler H. (2002). Sustained response to tumor necrosis factor alpha-blocking agents in two patients with SAPHO syndrome. *Arthritis & Rheumatism*.

[14] Yang Q., Xiang T., Wu Y., Ye E., He B., Bu Z. (2022). SAPHO syndrome with palmoplantar pustulosis as the first manifestation successfully treated with adalimumab. *Clinical, Cosmetic and Investigational Dermatology*.

[15] Garcovich S., Amelia R., Magarelli N., Valenza V., Amerio P. (2012). Long-term treatment of severe SAPHO syndrome with adalimumab. *American Journal of Clinical Dermatology*.

[16] Massara A., Cavazzini P. L., Trotta F (2006). In SAPHO syndrome anti-TNF-*α* therapy may induce persistent amelioration of osteoarticular complaints, but may exacerbate cutaneous manifestations. *Rheumatology*.

[17] Arias-Santiago S., Sanchez-Cano D., Callejas-Rubio J. L., Fernandez-Pugnaire M., Ortego-Centeno N. (2010). Adalimumab treatment for SAPHO syndrome. *Acta Dermato-Venereologica*.

[18] Vekic D. A., Woods J., Lin P., Cains G. D. (2018). SAPHO syndrome associated with hidradenitis suppurativa and pyoderma gangrenosum successfully treated with adalimumab and methotrexate: a case report and review of the literature. *International Journal of Dermatology*.

[19] Castellví I., Bonet M., Narváez J. A., Molina-Hinojosa J. C. (2010). Successful treatment of SAPHO syndrome with adalimumab: a case report. *Clinical Rheumatology*.

[20] Villaverde V., Mun˜oz-Fernández S., Hidalgo V. (1999). Acute anterior uveitis in a patient with sacroiliitis and acne conglobata. *Rheumatology*.

[21] Takeda A., Yui S., Hori J. *Ocular Manifestations of SAPHO Syndrome*.

[22] Yang Q., Zhao Y., Li C., Luo Y., Hao W., Zhang W. (2018 Jun). Case report: successful treatment of refractory SAPHO syndrome with the JAK inhibitor tofacitinib. *Medicine (Baltimore)*.

[23] Li B., Li G. W., Xue L., Chen Y. Y. (2020 Oct 6). Rapid remission of refractory synovitis, acne, pustulosis, hyperostosis, and osteitis syndrome in response to the Janus kinase inhibitor tofacitinib: a case report. *World Journal of Clinical Cases*.

